# We Need to Talk About Corruption in Health Systems

**DOI:** 10.15171/ijhpm.2018.123

**Published:** 2018-12-22

**Authors:** Eleanor Hutchinson, Dina Balabanova, Martin McKee

**Affiliations:** ^1^Department of Global Health and Development, London School of Hygiene and Tropical Medicine, London, UK.; ^2^Department of Public Health & Policy, London School of Hygiene and Tropical Medicine, London, UK.

**Keywords:** Corruption, Governance, Bribery, Absenteeism, Procurement

## Abstract

The health sector consistently appears prominently in surveys of perceived corruption, with considerable evidence that this has serious adverse consequences for patients. Yet this issue is far from prominent in the international health policy discourse. We identify five reasons why the health policy community has been reluctant to talk about it. These are the problem of defining corruption, the fact that some corrupt practices are actually ways of making dysfunctional systems work, the serious challenges to researching corruption, concerns that a focus on corruption is a form of victim blaming that ignores larger issues, and a lack of evidence about what works to tackle it. We propose three things that can be done to address this situation. First, seek consensus on the scale and nature of corruption. Second, decide on priorities, taking account the importance of the particular problem and the feasibility of doing something about it. Third, take a holistic view, drawing on a wide range of disciplines.

Some things are rarely discussed in public. Quite reasonably, most of us, if we are not among the growing number of social media celebrities, believe that the more intimate aspects of our personal relationships remain private. We can reasonably argue that they are of no concern to anyone else. But there are some things that take place in private, and which traditionally remained secret, that are at last being discussed openly because they do concern others. Sexual abuse and exploitation by those in positions of power exemplify such things. For too long, even though such behaviour was known about, it was tacitly accepted and few were sufficiently brave to talk about it. Those that did, especially if they were the victims, often suffered from doing so. They came to realise that actions that were, by any moral or ethical framework, wrong were simply accepted. That was the way things had always been, and would always be in the future. Quite simply, nothing could be done.

Yet something can be done. The #MeToo movement has empowered the victims of such abuse to speak out, holding the perpetrators to account. Now, it is the abusers who find themselves excluded. This does not mean that the problem has been solved. Few things are. Slavery was abolished in much the world more than 150 years ago but there are still hundreds of thousands of forced and indentured labourers and, in a few countries, slaves. But at least by talking about abuses of different kinds, it is possible to begin the process of changing things.

The #MeToo scandals are most closely identified with the arts world, although medicine is not immune.^1^ Yet we, in the health policy community, have our own dirty secret.^2^ It is something that we all know exists but we are often too polite to talk about it. It is corruption in the health sector. Too often, we spend long hours discussing why something that should work in theory does not work in practice, without asking who benefits from the status quo. Or why, despite investing in extra staff and facilities, many people still struggle to get the care they need, without asking where those staff are and what they are doing with the resources provided for them.

Our failure to confront this issue is all the more surprising given that surveys, such as those conducted by Transparency International, consistently find that the health sector is among the most corrupt in many countries. In its 2013 “corruption barometer” it reported that, in 42 of 109 countries surveyed, over 50% of citizens viewed their health systems as corrupt or very corrupt.^3^ Scholars have also developed detailed frameworks that describe the main manifestations of corruption in the health sector.^4,5^ We also know that this has consequences. It undermines the trust that underpins effective, equitable, and responsive healthcare.^6^ It also costs lives, with a 2011 study estimating that about 140 000 child deaths annually could be attributed to corruption.^7^ Yet when health ministers describe their achievements, both real and anticipated, at international meetings, while they will sometimes be frank and admit to specific failures, using them to discuss the lessons they have learned, they rarely discuss the role of corruption and the weaknesses of governance that often underlies it.

A new paper from a group of authors from North American universities makes the case for a renewed drive to tackle this issue. They argue that the Sustainable Development Goals (SDGs), which the governments of the world have committed to achieving by 2030, and including policies in a wide range of areas, could be leveraged to tackle corruption in the health sector.^8^ They showed how corrupt practices were likely to impede progress on several of the health-related SDGs, such as universal health coverage, but also how SDGs in other areas, such as SDG 16 (Peace, Justice and Strong Institutions) and 17 (Partnerships) offer means by which action might be mobilised.

As researchers working on an international project on corruption, in which we are focusing on the health sector, we have been reflecting on why it is that these conversations are so difficult to hold. We have identified five main reasons.

First, it is difficult to define corruption. Indeed, practices that are clearly corrupt are often described in other words. Even in one language, English, these include, for example, backhander, bribery, exploitation, extortion, fiddling, fraud, graft, nepotism, jobbery, payola, profiteering, racketeering, and skimming. Others, in languages other than English, have also gained international currency, even if their meaning has changed in the process. An example is baksheesh, which has evolved from its original meaning of charitable giving in Persian (‏بخشش‏)‎. “Dash”, used widely in West Africa, is believed to originate in the Portuguese word doação, or giving of gifts. Others are widely used in certain regions but have not spread widely elsewhere, such as the Urdu word rishwat (‏رشوت), used widely throughout the Indian sub-continent.

The United Nations Convention against Corruption does not try to define its subject, instead, simply listing a number of corrupt practices.^9^ The Cochrane Collaboration, in its review of measures effective in reducing corruption, did.^10^ It defines corruption as “The abuse or complicity in abuse, of public or private position, power or authority to benefit oneself, a group, an organization or others close to oneself; where the benefits may be financial, material or non-material.” This can take many forms, not all of which may be recognized as corruption by everyone.^4,11^ For example, irregularities in procurement of medical consumables and equipment, especially where these involved bribes, would probably be accepted by most people as corrupt. But what about the widespread practice of inducements, some of little monetary value, such as pens, by pharmaceutical sales representatives?^12^ There is considerable evidence that this is associated with increased prescribing of the brands concerned.^13^ Then there are informal payments, made to secure faster or higher quality treatment.^14-16^ Again, the corrupt nature may be obvious, but where is the dividing line from unsolicited tokens of gratitude? Theft of drugs or equipment from public facilities to be used in private ones is clearly corrupt, as it denies them to those in the public facilities. But what about absenteeism or late arrival or early departure from work in the same public facility, which also denies patients an essential component of care, the time of the health worker?^17,18^

Second, some practices that seem corrupt may actually be seen by those concerned, both providers of services and their beneficiaries, as the only means of enabling some fragile health systems simply to keep working. Health professionals may feel they have no alternative to bribe officials to obtain necessary authorisations to function. The recipients of the bribes are clearly corrupt, but what about those offering them? Informal payments extracted from those with the ability to pay may be channelled into the operation of the health facility rather than the pocket of the health worker, thereby allowing those who cannot afford payments to be treated. Should they be condemned for this? More generally, is there a danger that efforts to remove corruption without addressing the other weaknesses in the health system might threaten the delivery of care further and hurt the most vulnerable?

Third, how do we conduct research on corruption in ways that capture what is really happening? It is very easy for those involved in graft to shift blame to other, less powerful actors as corrupt and deflect attention from themselves. How does one engage in anti-corruption research without colluding with corrupt officials, for example when the researcher observes abusive or exploitative behaviour but seeks to maintain the access necessary to report it, a dilemma recognised in other settings where there are power imbalances?^19^

Fourth, is it even legitimate to study corruption? There is a view in some quarters that concerns about corruption divert attention from more important issues, such as the unequal distribution of global resources, itself a factor in the emergence and persistence of corruption. Some of those adopting this paradigm see a focus on corruption as a manifestation of the neoliberal attack on the state,^20^ noting how it was prioritized by development agencies in the 1980s during the Reagan-Thatcher era, when many public health systems were being dismantled.

Finally, we still know far too little about how to tackle corruption. Despite years of investment in good governance, levels of corruption remain high and, in some places, growing. The Cochrane review found no studies meeting their criteria that provided empirical evidence of effects of strategies to reduce corruption and only nine that could be used to describe the range of strategies that have been tried that could guide future evaluations of promising strategies, even if there is insufficient evidence so far.^10^

So, what can be done to address these problems and initiate a debate on corruption in health systems? A first step is to convene key stakeholders in the health system, including policy makers, health professionals, and managers, to seek agreement on the scale and nature of corruption in each health system. The challenges are, however, formidable. There will often be a considerable reluctance to speak openly, especially by those who are disempowered. Such individuals will need considerable support and, in some cases, protection if they are to expose corrupt practices.

Second, and once agreement has been reached on the problem, it will be necessary to prioritize action. Decisions should be guided by both the impact on the health system, and especially on vulnerable groups, and the existence of a potential remedy that has some feasibility of success. Some corrupt practices may not matter much while others might pose severe threats to health. Some may even be rational responses to a dysfunctional system. Sometimes, the choice will be helped by the opening of a window of opportunity. It will be very important to understand the reasons why corrupt practices thrive, constantly asking questions about who benefits in different ways and what are the underlying causes and the extent to which they can be changed. By understanding the incentives that operate, including the use of corrupt practices to overcome unnecessary structural and organizational barriers, it may be possible to develop pragmatic solutions that can command approval.^21^ Drawing on Kingdon’s work on policy streams, it is important to concentrate on what matters most and can most easily be addressed at a particular time.^22^

Third, as with many complex problems, it is essential to take a holistic view. Research on corruption in the health sector is often published in specialist anthropology and political economy journals and much less in the health literature. This means that it is largely absent from the main databases covering health systems, PubMed and EconLit, or if it is there it is difficult to find, perhaps because the term “corruption” is seen as judgmental and thus avoided in some cases. There is a clear need for a multi-disciplinary response, yet many countries where corruption is widespread lack traditions of multi-disciplinary working, which exacerbates their other problems related to limited research capacity. It is entirely understandable if researchers decide that they would rather study less contentious and, in some cases dangerous topics.

Finally, it is important that the research community sets out what it can offer. Zyglidopoulos et al identify four broad paths for research on corruption, in: (1) individuals, (2) organizations and industries, (3) different countries (largely from an economic development perspective), and (4) different cultural contexts (from an anthropological perspective).^23^ This work draws on a range of disciplines, including social psychology,^24^ criminology,^25^ development economics,^26^ and even media studies, such as that asking why a corrupt activity becomes a scandal.^27^ Now is an especially propitious time to do this, taking advantage of new opportunities, many of which fall within the umbrella of “big data,”^28^ using tools such as social network analysis.^29,30^

In writing this editorial, we hope to encourage a wider conversation about corruption in the health sector. This topic has failed to engage most global policy-makers, who seem to put it in the “too difficult” tray. Only a few groups, such as Transparency International, are campaigning for it to receive attention. Clearly, much could be done already, even without further research, where the political will exists. Yet, there is also a clear need for research, to ensure that policies adopted take account of the prevailing context and actually work, avoiding the risk of unintended consequences. A first step is to agree on a possible research agenda is lacking.

Above all, even if corruption is not yet spoken about publicly in the corridors of power, we know that it exists and is widespread. If we are to achieve the health-related SDGs, we must have that conversation within and among the many stakeholders within countries and the international community.

## Acknowledgements

This editorial is an expanded version of a blog by the authors on corruption, available at https://ace.soas.ac.uk/five-reasons-corruption-health/. It is an output of the SOAS Anti-Corruption Evidence (ACE) research consortium funded by UK Aid from the UK Government [Contract P0 7073]. The views presented in this publication are those of the authors and do not necessarily reflect the UK government’s official policies or the views of SOAS-ACE or other partner organisations. For more information on SOAS-ACE visit https://ace.soas.ac.uk.

## Ethical issues

Not applicable.

## Competing interests

Authors declare that they have no competing interests.

## Authors’ contributions

MM drafted the paper, which EH and DB revised.

## Authors’ affiliations

^1^Department of Global Health and Development, London School of Hygiene and Tropical Medicine, London, UK. ^2^Department of Public Health & Policy, London School of Hygiene and Tropical Medicine, London, UK.
